# Seroprevalence of Hepatitis B Virus and Compliance to Standard Safety Precautions among Scavengers in Ilorin Metropolis, Kwara State, Nigeria

**DOI:** 10.5696/2156-9614-8.19.180914

**Published:** 2018-09-10

**Authors:** Rauf O. Yusuf, Henry O. Sawyerr, Adedotun T. Adeolu, Lateefat M. Habeeb, Tawakalitu T. Abolayo

**Affiliations:** Department of Environmental Health Science, Kwara State University, Malete, Nigeria

**Keywords:** standard safety precautions, hepatitis B virus, scavengers, personal protective equipment, Ilorin metropolis

## Abstract

**Background.:**

Scavengers, due to the nature of their work, are at risk of various occupational hazards while sorting for materials for recycling and sale. They are exposed to blood borne infections such as hepatitis B virus (HBV) infection from injuries from sharps and contact with discarded materials due to non-compliance with standard precautions.

**Objective.:**

The present study assessed the prevalence of HBV and compliance with safety precautions among scavengers in Ilorin Metropolis, Kwara State, Nigeria.

**Methods.:**

A cross-sectional study was conducted among 236 scavengers (comprised of scavengers dealing with biomedical wastes and those that were not) using structured questionnaires during the first stage of the study and a second testing stage. Data were analyzed using the Statistical Package for the Social Sciences (SPSS) software version 20.0 for descriptive and inferential statistics at a 5% level of significance.

**Results.:**

The prevalence of HBV infection among the scavengers was found to be 17.4%, indicating that scavengers are at high risk of HBV infection. There was a significant difference in the prevalence of HBV among scavengers that handled biomedical wastes and those that did not. The majority of the scavengers (74.2%) did not have knowledge of or use standard precautions such as personal protective equipment (PPE). The level of injuries was considerably high and the treatment of injuries among scavengers illustrated their lack of awareness of the hazards involved in their occupations, as the majority of respondents (51.3%) engaged in self-treatment of occupational injuries.

**Conclusions.:**

The probable pathway for virus transmission was waste handling, especially biomedical waste, which is mostly handled with bare hands without standard safety precautions. Vaccination against HBV, proper personal hygiene practices, regular training in occupational safety, monitoring by regulatory agencies and inclusion of scavengers in a mandatory health insurance scheme are recommended to control the risk of HBV infection among scavengers.

**Informed Consent.:**

Obtained

**Ethical Approval:**

This study was approved by the Kwara State Ministry of Health Ethical Review Committee. Permission was also granted by the scrap dealers association through the Kwara State Environmental Protection Agency that oversees issues relating to the environment and public health in the state.

**Competing Interests.:**

The authors declare no competing financial interests.

## Introduction

Hepatitis is an inflammatory condition of the liver and viral hepatitis is a conventional term used to denote hepatitis caused by hepatotrophic viruses (A–G). High prevalence of these viruses, especially hepatitis B, has been reported in Nigeria.^1^ Hepatitis B virus (HBV) accounts for about 1 million deaths worldwide annually.^2^ The pooled prevalence of HBV from Nigerian studies between 2000 and 2013 was 13.6% for adults and 11.5% for children.^3^ Variance in the seroprevalence of HBV in Nigeria was based on the type of screening method used (enzyme-linked immunosorbent assay (12.3%), immunochromatography (17.5%) and DNA polymerase chain reaction (13.6%)) and the accuracy of the instrument.^3^ Hepatitis B virus causes liver cirrhosis and can be contracted through transfusion of unscreened blood and its products, use of inadequately sterilized and contaminated instruments and sharing of sharp instruments in traditional or cultural practices.^2^,^4^,^5^

Hepatitis B infection is also recognized as an occupational health hazard for health-care practitioners.^6–10^ Health-care workers have a 3- to 5-fold higher prevalence of HBV than the general population, with surgeons and dentists at greatest risk.^11^ Health risks of waste scavenging are numerous, but are broadly classified into occupational and environmental hazards.^12^,^13^ Occupational risks include biological pathogens such as parasites and bacteria related to the gastro-intestinal tract. This can be passed from hands to the mouth. Hospital waste often constitutes part of the waste.^14^ This can be hazardous in terms of biological and chemical contamination, including exposure to used syringes, dressings, discarded medicine and sometimes body parts. Industrial waste may include toxic materials such as heavy metals and has associated health effects. Sharp objects can cause cuts which, in turn, may lead to tetanus or other infections.^15^ Environmental risks include the risks posed by scavenging activities to the environment. The waste they collect can contaminate the air, water and soil of the environment in which they live. They are, therefore, often doubly exposed to the environmental hazards listed above.^16^,^17^ Other risks include sexual harassment from peers, hounding by police and local residents, and competition over waste materials that sometimes lead to violent clashes among scavengers.^12^,^18^

Scavengers, sometimes referred to as waste pickers, make a living by selling materials they collect from dumpsites, bins and from along roadsides. Typically, this waste comes from domestic, industrial, biomedical and commercial sources.^12^,^19^ Throughout cities in developing countries, varying numbers of poor individuals survive by recovering materials to sell for reuse or recycling, as well as diverse items for their own consumption from the waste stream.^19^,^20^ Most studies report that human scavengers constitute poorer segments of the population in developing countries.^21^ It has been estimated that up to 2% of the population in developing countries survives by recovering materials from waste.^22^ However, scavengers are not refuse workers and they are not concerned with waste management. They enter into trade for socioeconomic reasons and their relationship with waste is as a resource, as they only collect those materials for which there is market, including hospital waste.^14^,^16^ In Nigeria, the importance of the role of waste scavengers in the waste recycling process cannot be over-emphasized. Scavengers informally make a significant contribution towards the provision and separation of recyclables for recycling industries.^23^ Scavenging offers a form of employment to a significant population of youth from the slum areas of cities that otherwise would have no means of livelihood.^24^

Handling of wastes among scavengers has been of great health concern, especially in developing countries like Nigeria where scavengers are exposed to occupational health and safety risks as a result of unsafe handling of waste materials and lack of protective clothing and equipment.^21^,^25^ Due to the dangerous environment in which they work, scavengers often suffer temporary injuries that may become permanent.^19^ Individuals may lose their source of livelihood as a result of an accident or injury that in the developed world would be considered relatively minor and readily treatable. Minor cuts, for example, can quickly become infected in unhygienic and contaminated working conditions. Infection might prevent a waste scavenger from working for a period of time, but it might also lead to the loss of a limb and consequent permanent loss of livelihood, as well as death.^4^,^13^ Waste scavengers are constantly in contact with the wider public during the course of their daily jobs.^5^ Their health status is therefore of public health concern, as they could be potential pathways for the transmission of various communicable diseases to the general public.^21^

**Abbreviations**
*HBsAg*: Hepatitis B surface antigen
*HBV*: Hepatitis B virus
*PPE*: Personal protective equipment

Awareness of the risk of hepatitis B virus is generally low in Nigeria, especially among waste scavengers, due to their socio-economic status and low level of education.^1^,^21^ Proper awareness of the risks posed by HBV could increase vaccination rates and compliance with safety precautions.^4^,^21^ Compliance with standard precautions reduces the risk of exposure to blood and body fluids.^26^,^27^ Knowledge of standard precautions may be influenced by the types of training to which workers are exposed.^28^ These standard precautions include hand washing, use of barriers (gloves, gown, face mask, boot etc), and careful use of proper equipment and clothing.^29^,^30^ Of these precautions, hygiene, vaccinations and barriers are the most important.^31^ The present study assessed the seroprevalence of HBV and compliance to safety precautions among waste scavengers in Ilorin Metropolis, Kwara State, Nigeria.

## Methods

The city of Ilorin lies on latitude North 8°30' and longitude East 4° 35' near the southern fringe of the savannah and forest zone. It had a population of 777,667 in the 2006 census. It is surrounded by a wall about 10 miles in circumference and as high as 20 feet in some places. A large part of the province is located on grass plains with undulating landscapes which are well watered and highly agricultural. By the southern Nigeria provincial borders, at an elevation of 1,500 feet, there is a watershed with a river generally running from west to east and flowing into the River Niger. The ecology of the region plays an important role in people's decisions to settle in a particular area. It has a mean annual rainfall of 1,318 mm (51.9 inches), which allows inhabitants to practice arable farming. The mild climate has also attracted northern pastoralists to the region. Ilorin city is the commercial and administrative center of Kwara State. It is made up of four local government areas (Ilorin South, Ilorin West, Ilorin East and Asa).

### Study design

**Figure 1:**
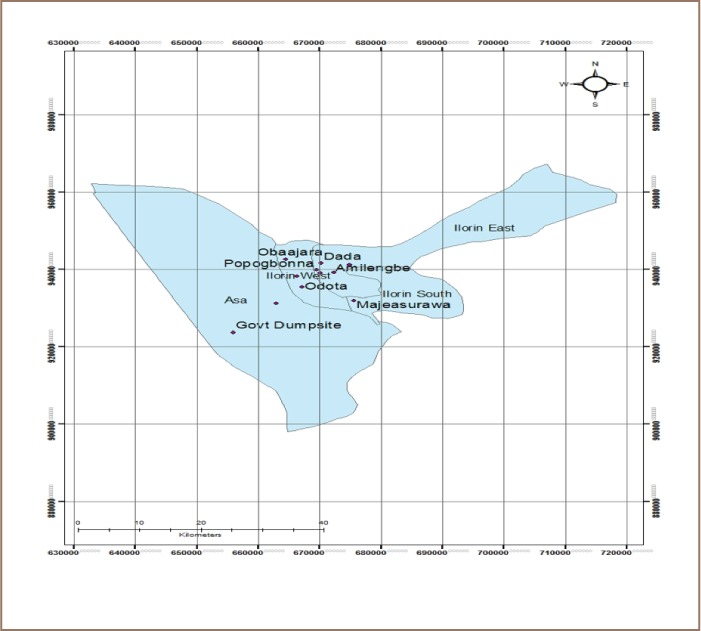
Map of Ilorin Metropolis showing selected major scrap dealer locations. Source: Author field survey (2017)

The present study employed a cross-sectional study design to assess the seroprevalence of HBV and compliance to safety precautions among scavengers in Ilorin Metropolis, Kwara State, Nigeria. The study population was comprised of scavengers working with the major registered scrap dealers and in the dumpsites in Ilorin metropolis, Kwara State, Nigeria *(Figure 1).*

### Sampling

Waste scavengers were randomly selected from the available scrap dealers which buy recycling materials. Out of twenty-five registered scrap dealers, eleven were systematically selected to reflect sampling in all of the zones of Ilorin metropolis and dumpsites. One of the criteria for the present study was that study participants were scavengers working with major scrap dealers scavenging for commercial purposes and registered with the scrap dealers association. The total sample size of 236 was arrived at using Fisher's formula.^32^ The sampling technique employed in the present study was purposive, as only those identified as waste scavengers working with major scrap dealers and in dumpsites were interviewed and screened for HBV. Scavengers were selected from government dumpsites as well as registered scrap dealers through the Kwara State Environmental Protection Agency as well as the Association of Scrap Dealers, Ilorin Branch. Respondents were administered questionnaires and gave consent before proceeding to the second stage of the study (hepatitis B surface antigen (HBsAg) screening).

### Data collection

A semi-structured questionnaire was administered to elicit information on the socio-demographics and occupational hazards faced by scavengers. Their knowledge of HBV, use of protective clothing and equipment during work activities, treatment of injuries and training in waste handling were examined. A geographical positioning system (GPS) was used to geo-code the location of major scrap dealers where scavengers converge to sell their materials. The instrument was pre-tested among 24 scavengers in Amoyo, Ifelodun in Kwara State, which is similar to the study area and has a sizeable number of scavengers. The Cronbach's alpha reliability test coefficient was 0.84, signifying good reliability. Each question was translated into local languages (i.e. Yoruba and Hausa) for those that could not read English, to help the respondents give true and accurate answers.

### Sample collection

Blood samples were collected from subjects with the help of a medical laboratory scientist using intravenous needles. The needles and syringes used for the collection of blood samples were dried and sterile. Three (3) ml of venous blood was aseptically drawn from the antecubital vein of participants into a plain bottle and allowed to clot at room temperature; the sample was then spun for 5 minutes at 2500 rpm in a bench centrifuge to obtain serum. The serum obtained was tested for HBsAg antibodies using a Diaspot rapid diagnostic test strip. The rapid diagnostics test was chosen based on the immune chromatographic principle and its accuracy.^33^

### Rapid diagnostics test

The Diaspot rapid diagnostic test is used to qualitatively detect the presence of HBsAg in serum or plasma specimens. The test utilizes a combination of monoclonal and polyclonal antibodies to selectively detect elevated levels of HBsAg in serum or plasma. The membrane is pre-coated with anti-HBsAg antibodies on the test line region. During testing, the serum or plasma specimen reacts with particles coated with anti-HBsAg antibody. The immunochromatographic reaction was allowed to take place within a few minutes and the result read at exactly 15 minutes. The mixture migrates upward on the membrane chromatographically by capillary action to react with anti-HBsAg antibodies on the membrane and generates a colored line. The presence of the colored line in the test region indicates a positive result, while its absence indicates a negative result. To serve as a procedural control, a colored line will always appear in the control line region, indicating that the proper volume of specimen has been added and membrane wicking has occurred.^34^

**Figure 2:**
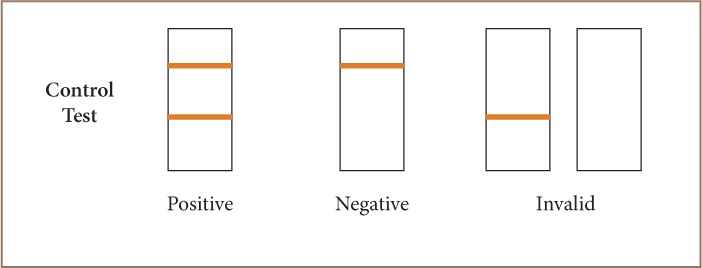
Rapid diagnostic test for hepatitis B surface antigen

The manufacturers' instructions were strictly followed in the performance of these tests. The HBsAg assay has a manufacturer-reported diagnostic specificity, sensitivity and accuracy of >99.0%, 97.0% and 98.5%, respectively. The test strips, serum or plasma specimens were allowed to equilibrate to room temperature (15–30°C) prior to testing. The test device was placed on a clean, level surface and 60 μl of serum or plasma was added to the sample well of the device. The sample was rehydrated and mixed with the red colloidal gold conjugate, which flowed into the membrane. After 10–15 minutes, red line(s) appeared which were read for the results of the test. The results of the test were reported as positive, negative or invalid (*Figure 2*). For each invalid test, the test procedure was reviewed and the test repeated with a new strip. A positive HBsAg test was considered evidence of HBV infection (chronic carrier state or infection) and used to calculate prevalence.^34^

### Data analysis

Data were analyzed using SPSS version 20. Data were analyzed using descriptive statistics such as mean and standard deviation. The chi-square (x2) was used for univariate analysis for the comparison of the prevalence of HBV markers in study subgroups (whether or not scavengers were exposed to biomedical wastes).

### Ethical approval

This study was approved by the Kwara State Ministry of Health Ethical Review Committee. Permission was also granted by the scrap dealers association through the Kwara State Environmental Protection Agency that oversees issues relating to the environment and public health in the state. Informed consent was obtained from each respondent before administration of the questionnaires and testing, having clearly stated that participation in the study was voluntary and individuals may decide not to further participate in the research at any time.

## Results

The mean age was 26.9 years and the majority (37.3%) of the respondents were between 21–30 years of age. More than half (59.3%) of the respondents were married and most were male (94.1%). The majority (36.4%) had attended primary school and (34.3%) had no formal education. The distribution of respondents according to ethnicity showed that the majority (84.7%) were Hausa *(Table 1).* It was noted that 228 respondents (96.6%) had no training in waste handling or occupational health *(Table 2).*

**Table 1:** Demographic Characteristics of Respondents

**Variables**		**Frequency**	**Percentage**
***Sex***	**Male**	222	94.1
	**Female**	14	5.9
***Age***	**10–20 years**	63	26.7
	**21–30 years**	88	37.3
	**31–40 years**	56	23.7
	**41 years or older**	29	12.3
***Marital Status***	**Single**	96	40.7
	**Married**	140	59.3
***Tribe***	**Hausa**	200	84.7
	**Yoruba**	32	13.6
	**Igbo**	4	1.7
***Religion***	**Christian**	13	5.5
	**Islam**	221	93.6
	**Other**	2	0.8
***Highest Level of Education***	**No formal**	81	34.3
	**Primary**	86	36.4
	**Secondary**	65	27.5
	**Tertiary**	4	1.7

**Table 2:** Awareness of the Risks and Standard Safety Practices by Scavengers

**Variables**		**Frequency**	**Percentage**
***Works with biomedical waste***	**Yes**	124	52.5
	**No**	112	47.5
***Belief in the Practice of Burning Waste for Scrap Metals***	**Bad**	154	65.3
	**Good**	82	34.7
***Belief of Effect of Burning Wastes***	**Can cause disease**	186	78.8
	**Cannot cause disease**	50	21.2
***Awareness of PPE***	**Yes**	54	22.9
	**No**	182	77.1
***Use of PPE***	**Yes**	61	25.8
	**No**	175	74.2
***Frequency of Injuries at Work***	**Three times a week**	52	22.0
	**Once/twice a week**	169	71.6
	**No injury**	15	6.4
***How Injuries are Treated***	**Chemists**	40	16.9
	**Clinic**	32	13.6
	**Self-treatment**	121	51.3
	**Leave to heal**	43	18.2
***Awareness of HBV***	***Yes***	20	8.5
	***No***	216	91.5
***Vaccinated against HBV***	***Yes***	2	0.8
	***No***	234	99.2
***Trained on Waste Handling***	***Yes***	8	3.4
	***No***	228	96.6

**Abbreviations**: HBV, hepatitis B virus; PPE, personal protective equipment

More than half (52.5%) of respondents reported scavenging from bio-medical waste. About sixty-five percent of scavengers (65.3%) reported that burning of wastes to remove valuable scrap metal is bad for the environment. With regard to awareness of the effect of waste burning on disease risk, most of the respondents (78.8%) believed that waste burning can increase the risk of getting some diseases. It was noted that the majority (77.1%) of the scavengers did not understand the importance and usefulness of personal protective equipment (PPE). One-quarter (25.8%) of the scavengers used some form of PPE while working. The rate of injuries among the respondents during the course of their job was assessed and almost three-quarters (71.6%) reported getting injured once or twice a week. The majority (51.3%) of the scavengers practiced self treatment. Almost all of the scavengers (91.5%) reported no awareness of HBV. With regard to vaccination against HBV, only two (0.8%) of the scavengers had been vaccinated *(Table 2).*

The respondents were questioned about common ailments and malaria was reported to be the most frequent ailment among the three most common diseases *(Table 3).* More than half (54.7%) had experienced malaria more than twice in the last six months, and 19.9% and 21.6% experienced the same for dysentery and cough, respectively.

**Table 3:** Health Status of Scavengers

	**Frequency**	**Percentage**
**Malaria**	129	56.8
**Dysentery**	47	20.7
**Cough**	51	22.5
**Malaria [?]**	0	0

The results of the hepatitis B test showed that 195 (82.6%) respondents were negative, while 41 (17.4%) respondents were positive to the virus, i.e. seropositive to HBsAg *(Table 4).*

**Table 4:** Prevalence of Hepatitis B Virus Infection Among Respondents

**Variables**		**Frequency**	**Positive**	**Negative**	**Chi square**
***HEPATITIS B VIRUS***			41(17.4)	195 (82.6)	
***Gender***	**Male**	222	38	184	4.682^*^
	**Female**	14	5	9	
***Age***	**<=20 years**	63	4	59	
	**21–30 years**	88	12	76	6.248^*^
	**31–40 years**	56	16	40	
	**>=41 years**	29	9	20	
***Marital Status***	**Single**	96	7	89	8.341^*^
	**Married**	140	35	105	
***Tribe***	**Hausa**	200	34	166	
	**Yoruba**	32	6	26	
	**Igbo**	4	1	3	
***Religion***	**Christian**	13	3	10	
	**Islam**	221	38	183	5.924^*^
	**Traditional**	2	0	2	
***Level of Education***	**No formal**	81	21	60	
	**Primary**	86	12	74	6.235+
	**Secondary**	65	8	57	
	**Tertiary**	4	0	4	
***Type of Waste Scavenging***	**Biomedical (inclusive)**	124	37	87	8.352^*^
	**Non-biomedical**	112	4	108	
***Use of PPE***	**Yes**	61	04	57	6.921^*^
	**No**	175	37	138	
***Vaccination against HBV***	**Yes**	2	0	2	3.281^*^
	**No**	234	41	193	

**Abbreviations:** HBV, hepatitis B virus; PPE, personal protective equipment

+ = **p>0.05;**

^*^ = **p<0.05**

The results showed that there were significant differences in the prevalence of Hepatitis B virus among scavengers with regard to age, sex, marital status, use of PPE, vaccination or type of waste scavenging (those who work with bio-medical wastes and those who do not) *(Table 4).*

## Discussion

The results indicated that most scavengers (37.3%) in Ilorin metropolis are between 21–30 years of age. This is in contrast with findings indicating that the majority of the scavengers in Ilorin (61%) are teenagers, but in agreement with a study that reported that 54% of scavengers in Mubi, Adamawa State were between 20–29 years.^21^,^35^ The results showed that scavenging in Ilorin metropolis is a male dominated profession. This is in conformity with the findings on scavengers in Malaysia that reported that 75% of the respondents were male.^2^ A study of scavenging in Kano State, Nigeria found that male youth dominate the profession.^36^ The majority of scavengers (36.4%) had a primary school education and very few (1.7%) had received tertiary education. This is in agreement with a study by Adeyemi *et al.* that found that the majority of scavengers in Ilorin possessed a primary school education.^34^ However, it differed from a study by Mustafa who found that few scavengers had a formal education.^36^ The length of time that scavengers spent doing their work was examined and 45.8% spent more than 7 hours per day at the dumpsite or scavenging recyclables to sell. This is in agreement with a study by Mustafa that found that the majority (53%) of scavengers spent between 6 – 12 hours per day scavenging.^36^

Hepatitis B is a disease of public health importance endemic in many parts of the world which causes both acute and chronic infection with significant complications and sequelae. The seropositivity for HBsAg in the present study was 17.4%. This prevalence is the range of most studies carried out in Nigerian children with prevalences ranging from 4.1% to 44.7%.^3^,^37–40^ A prevalence of 12.7% was reported among the parturients in the University of Ilorin Teaching Hospital, Nigeria.^41^ There was a significant association between increasing age and positivity to HBsAg in the present study. This followed a trend reported in Benin City and Maiduguri, Nigeria, which suggested that vertical transmission may not play a major role in the spread of HBV infection in Nigeria.^42^,^43^ It was also evident that HBV seropositivity increased progressively with age, and this was attributed to increasing exposure to the virus by a significant proportion of the scavengers.

It has been reported that high risk behaviors such as scarification and tattooing might be responsible for the high rates of positive HBV infection among respondents after controlling for confounding factors.^21^ Body piercing is not common among Nigerians, especially the social strata in which scavengers fall. A study conducted on the prevalence of HBV among students and non-teaching staff of Nile University in Abuja found that those who practiced safe sex had lower prevalence of HBV infection than those who did not, and the difference was statistically significant (p<0.001). Likewise, those who were not sexually active recorded a significantly lower prevalence (all sexually inactive individuals in this study were negative) compared to those who were sexually active (p<0.001). Hence, unsafe sex could also be a contributing factor in exposure to HBV.^44^

In Nigeria, international policies specifying that waste generators are responsible for the proper management, treatment and disposal of waste have yet to be implemented. The notion that waste management is the sole responsibility of government authorities means that waste generators do not appreciate the negative impact of improper waste disposal, especially the hazardous nature and disease transmission characteristics of some wastes.^21^ Most healthcare facilities dispose of their waste via waste contractors. Waste contractors working within the State are poorly trained and handle different types of waste regardless of their source. Most hospitals and medical facilities do not segregate or treat wastes at the source, hence wastes are co-mingled. Scavengers source their recyclables from dumpsites and other illegal dumping areas and since there is no dedicated disposal site or dumpsite for bio-medical wastes, scavengers source recyclables from any waste brought to the dumpsites, which often contains needles, scalpel blades or body parts that could cause infection.

Assessment of the prevalence of hepatitis B in Nigeria reported a mean prevalence of 10.7%, confirming that HBV infection is highly endemic in Nigeria.^45^ This high prevalence calls into question the effectiveness of the Nigerian HBV vaccination program. The prevalence of the hepatitis B virus appears to be higher among scavengers as it is considerably higher than the overall prevalence rate of hepatitis B infection among students and non-teaching staff of Nile University, Nigeria (6.5%).^44^ The rate of vaccination against HBV remains low even though vaccines are available and incorporated into the national immunization program.^46^,^47^ This finding is similar to those of studies conducted in Nigeria which have reported a low/poor vaccination status among healthcare workers who are at higher risk of occupational exposure to HBV.^48–50^

**Figure 3:**
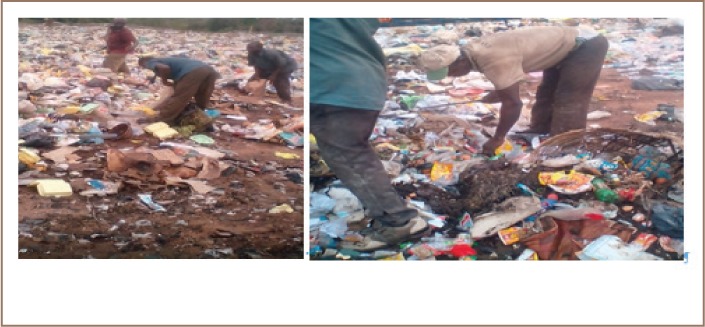
Scavengers at work at Eyenkorin dumpsite in Asa Local Government area. Source: Author field survey, (2017).

In the present study, scavengers had poor knowledge of universal standard precautions and did not fully appreciate their occupational risk regarding hepatitis B infection. Non-compliance with these precautions could be due to lack of knowledge of occupational hazards, perceived reduction in dexterity when wearing gloves, and the absence of penalties for failure to use PPE. The majority of the scavengers lacked training on proper waste handling procedures, especially with regard to biomedical waste *(Figure 3).* The present study also showed that knowledge and implementation of precautions to prevent infection among scavengers was low. There is a need for continuous and consistent training on waste handling and compliance with universal standard precautions, including use of PPE and vaccination. Studies have reported significant improvement ranging from 48% to 74% in compliance with standard precautions after an educational symposium and after a 30-minute educational program.^23^,^51–52^ Greater availability of training and awareness programs could increase compliance with standard precautions for waste handling.^4^,^53^

## Conclusions

The present study showed that there is high seroprevalence of HBV infection among waste scavengers, indicating possible pathways of virus transmission through waste handling, which is usually carried out with bare hands, and a lack of hygiene and occupational safety measures during waste management activities. Efforts should be made to provide scavengers with PPE and thorough instructions on its use. In addition to vaccination against HBV, educational campaigns and regular training on occupational health and safety programs and health surveillance should be instituted for all waste workers with an emphasis on good work practices, immunization and personal hygiene practices to prevent HBV infection among members of this occupational group. It is the responsibility of waste generators to ensure that waste is properly segregated, treated and transported to designated sites for proper disposal and not disposed of in municipal/general waste sites. Scrap dealers should refuse to accept items coming from healthcare facilities if waste is not properly treated.

The present study illustrates the need to build the capacity of scavengers with regard to standard precautions either through regular training workshops and seminars by environmental health officers from the State Ministries of Environment and Health and the Kwara State Environmental Protection Agency. This is the most effective and long-lasting means to improve scavengers' knowledge and foster compliance with standard precaution measures. Scavengers should be integrated into municipal waste management structures, provided with safer tools and enrolled in a compulsory health insurance scheme sponsored by the government. The local government should institute compulsory HBV screening of all scavengers in Ilorin metropolis and those that test negative should be vaccinated, while those that test positive should receive free medical treatment.
